# TnSeq of *Mycobacterium tuberculosis* clinical isolates reveals strain-specific antibiotic liabilities

**DOI:** 10.1371/journal.ppat.1006939

**Published:** 2018-03-05

**Authors:** Allison F. Carey, Jeremy M. Rock, Inna V. Krieger, Michael R. Chase, Marta Fernandez-Suarez, Sebastien Gagneux, James C. Sacchettini, Thomas R. Ioerger, Sarah M. Fortune

**Affiliations:** 1 Department of Immunology and Infectious Diseases, Harvard T.H. Chan School of Public Health, Boston, Massachusetts, United States of America; 2 Department of Pathology, Massachusetts General Hospital, Boston, Massachusetts, United States of America; 3 Department of Biochemistry and Biophysics, Texas A&M University, College Station, Texas, United States of America; 4 Department of Medical Parasitology and Infection Biology, Swiss Tropical and Public Health Institute, Basel, Switzerland; 5 University of Basel, Basel, Switzerland; 6 Department of Computer Science, Texas A&M University, College Station, Texas, United States of America; 7 Ragon Institute of MGH, MIT, and Harvard, Cambridge, Massachusetts, United States of America; 8 Broad Institute of MIT and Harvard, Cambridge, Massachusetts, United States of America; New Jersey Medical School, UNITED STATES

## Abstract

Once considered a phenotypically monomorphic bacterium, there is a growing body of work demonstrating heterogeneity among *Mycobacterium tuberculosis* (Mtb) strains in clinically relevant characteristics, including virulence and response to antibiotics. However, the genetic and molecular basis for most phenotypic differences among Mtb strains remains unknown. To investigate the basis of strain variation in Mtb, we performed genome-wide transposon mutagenesis coupled with next-generation sequencing (TnSeq) for a panel of Mtb clinical isolates and the reference strain H37Rv to compare genetic requirements for *in vitro* growth across these strains. We developed an analytic approach to identify quantitative differences in genetic requirements between these genetically diverse strains, which vary in genomic structure and gene content. Using this methodology, we found differences between strains in their requirements for genes involved in fundamental cellular processes, including redox homeostasis and central carbon metabolism. Among the genes with differential requirements were *katG*, which encodes the activator of the first-line antitubercular agent isoniazid, and *glcB*, which encodes malate synthase, the target of a novel small-molecule inhibitor. Differences among strains in their requirement for *katG* and *glcB* predicted differences in their response to these antimicrobial agents. Importantly, these strain-specific differences in antibiotic response could not be predicted by genetic variants identified through whole genome sequencing or by gene expression analysis. Our results provide novel insight into the basis of variation among Mtb strains and demonstrate that TnSeq is a scalable method to predict clinically important phenotypic differences among Mtb strains.

## Author summary

Tuberculosis, caused by the bacterium *Mycobacterium tuberculosis* (Mtb), remains a serious global health problem, causing ~1.5 million deaths a year world-wide. Like other bacterial pathogens, diversity among strains of Mtb contributes to differences in infection outcomes, vaccine efficacy, and response to antibiotic treatment. Currently, the important genetic differences that contribute to variation among Mtb strains remain poorly understood. In this study, we applied a functional genomics technique called TnSeq to a panel of Mtb clinical strains to investigate the genetic basis of strain diversity. We identified a number of genes that are differentially required for growth in culture among these strains. Some of these genes are involved in the response to antibiotics, including the first-line antitubercular agent isoniazid and a novel antitubercular drug currently in development. We found that the genetic differences between strains uncovered by TnSeq predicted responses to these antibiotics. Our results demonstrate the utility of TnSeq for identifying clinically relevant differences among Mtb strains.

## Introduction

A hallmark of infection with *Mycobacterium tuberculosis* (Mtb) is the high degree of variability in disease course and response to therapy. This heterogeneity in Mtb infection outcome and treatment response has traditionally been attributed to variability in host determinants. Yet it is increasingly apparent that meaningful differences exist among Mtb strains in features that impact immunogenicity, virulence, and response to antibiotic treatment [1–4]. Despite the potential consequences of strain variation for the development of new diagnostics, drugs, and vaccines, predicting and defining the causal genetic determinants of biologically important phenotypic variation between strains remains a challenge.

Our understanding of strain heterogeneity has been vastly improved through the advent of affordable next-generation sequencing technologies. This has facilitated the whole genome sequencing (WGS) of thousands of Mtb clinical strains and uncovered numerous sequence variants including single nucleotide polymorphisms (SNPs), insertions-deletions (in-dels), large sequences polymorphisms (LSPs), and insertion element transpositions [5–11]. Sequence variation is used as a proxy for phenotypic diversity, yet the functional consequences of most polymorphisms in the Mtb genome are not known. The field’s ability to predict phenotype from genotype is most well developed for resistances to clinically important first and second line drugs, where large population-based studies have been successful in identifying genetic determinants of antibiotic resistance [6,8,11–13]. Yet extending such analyses to predict responses to new antibiotics or to predicting more complex phenotypes such as virulence remains difficult. In the setting of antibiotic development, sequence conservation is often used to indicate conservation of gene function, but does not reflect capacity for the emergence of resistance or plasticity in the underlying genetic and cellular networks that may create strain-to-strain differences in antibiotic efficacy. Thus, there is a need for systematic and high-throughput methods to predict phenotypic variation among Mtb strains, especially for clinically relevant phenotypes such as antibiotic susceptibility, likelihood of drug resistance, and virulence.

TnSeq, genome-wide transposon mutagenesis coupled with next-generation sequencing, has emerged as a high-throughput approach to define the contributions of genes and genetic networks to microbial phenotypes [14–21]. TnSeq experiments reveal the fitness cost of gene disruption, and thus can identify both global and context-specific genetic requirements. In Mtb, TnSeq has been used to identify genes and pathways required across a variety of contexts, including defining the genes essential for *in vitro* growth, which has provided a roadmap for antibiotic development [17–19].

Most TnSeq studies are performed on reference strains, including those in Mtb, which, to date, have been conducted exclusively with laboratory-adapted reference strains. In the published literature, a small number of TnSeq studies in other pathogens have performed pairwise comparisons between a reference strain and a single clinical isolate to define differences in the genetic requirements for growth *in vitro* or under antibiotic stress [14,22,23]. These studies have identified strain-to-strain differences in requirements for key pathways including aspects of central metabolism. However, it is unclear how representative these findings are since the choice of a single clinical isolate is relatively arbitrary. Comparisons across larger panels of non-reference (clinical) strains have not been performed in part because there has been no systematic methodology to analyze TnSeq data across multiple genetic backgrounds. Differences in genome structure and gene content between strains create significant challenges for TnSeq analysis since existing approaches rely heavily on mapping sequencing reads to a reference genome. Gene deletions can result in false gene essentiality calls, gene duplications may result in false gene non-essentiality calls, and sequence variants that alter transposon insertion sites can create more subtle errors. Thus, an analytic approach that permits rigorous statistical analysis in the setting of genomic variation is needed.

In this work we developed a pipeline to use TnSeq to comprehensively define genetic requirements for *in vitro* growth across a diverse panel of nine Mtb strains, with the goal of predicting strain-specific differences in the requirements for genes that are the target of antibiotic development. In contrast to sequence data, which has limited phenotypic predictive power without prior knowledge about causal variants, the genetic requirements identified through TnSeq directly reflect the cost of gene disruption on microbial growth. Therefore, differences in genetic requirements identified from our TnSeq data allowed us to successfully predict differences between strains in the responses to both a widely used and a novel antibiotic. Importantly, these differences could not be readily predicted from WGS or gene expression data. Our work demonstrates the power of high-throughput mapping of genetic requirements via TnSeq to uncover clinically relevant phenotypic differences between Mtb strains.

## Results

### High-density transposon libraries of genetically diverse Mtb clinical strains

It has become clear from a growing number of *in vitro*, animal model, and epidemiological studies that important phenotypic differences exist among Mtb strains [24]. Yet how to predict phenotypic variation among strains systematically is unclear. Because TnSeq provides a direct read out of the genes required for growth under a given condition, we hypothesized that TnSeq might be a robust method to address this gap. We therefore sought to use TnSeq to define differences in the genetic requirements for *in vitro* growth across a panel of genetically diverse Mtb clinical strains.

**Fig 1:**
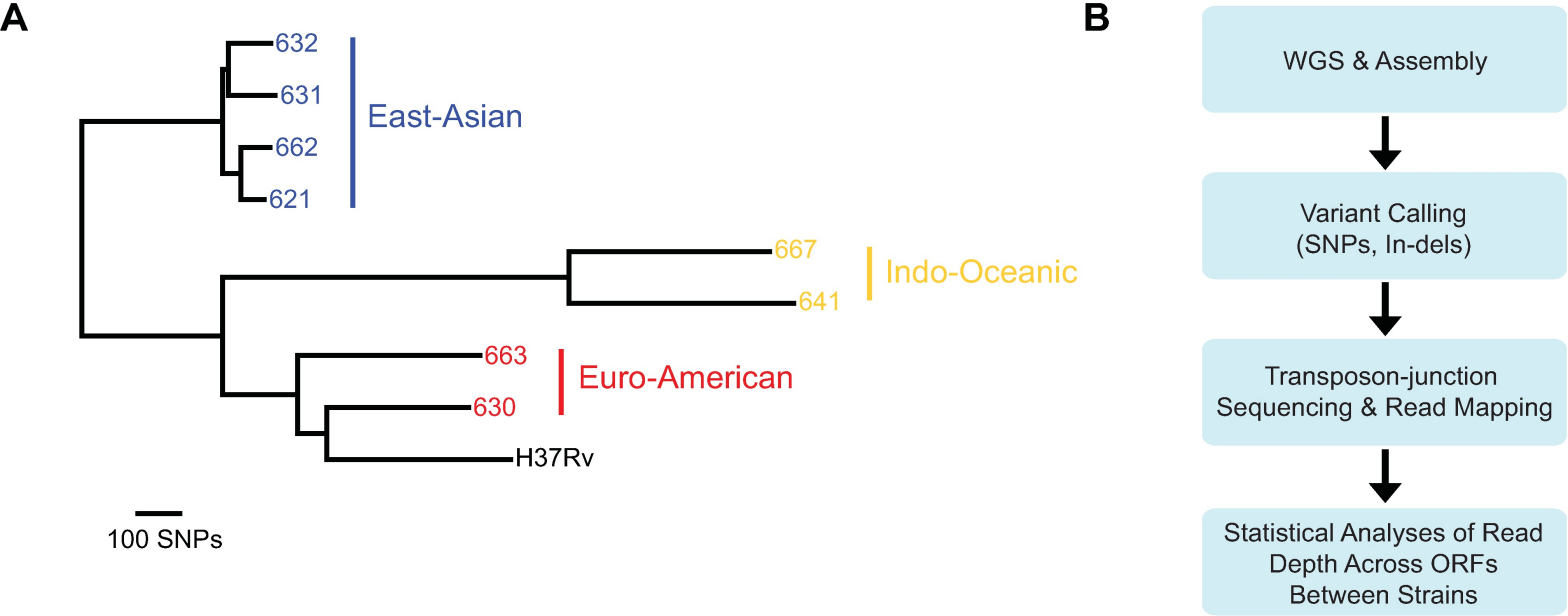
A genetically diverse panel of *M*. *tuberculosis* clinical strains subjected to TnSeq. (A) Maximum parsimony tree constructed from SNPs identified through WGS of each clinical strain. The reference strain H37Rv is included. (B) Schematic illustrating the analytic pipeline.

We focused on a panel of 8 Mtb clinical strains (Fig 1A). These strains had previously been classified by large sequence polymorphisms (LSP), a low-resolution method of genotyping, as belonging to three of the major Mtb phylogenetic lineages [25], and the most prevalent lineages currently in circulation: Euro-American, East Asian, and Indo-Oceanic (Fig 1A). To define the genetic requirements for *in vitro* growth for these strains, we performed transposon mutagenesis using the modified *Himar1* transposon [26] and included the reference strain H37Rv as a control. Transposon libraries were generated in biological duplicate for each clinical strain, and in triplicate for the control strain, H37Rv. The libraries were then subjected to transposon-junction sequencing [27].

Because interpretation of TnSeq data relies on the accurate mapping of transposon-junction reads to the genome of the mutagenized strain, data analysis can be confounded by sequence variants. To account for the genetic variation in our panel of clinical strains, we performed WGS on each strain, followed by reference-based assembly, annotation, and variant calling (Fig 1B, Materials and Methods). WGS revealed that, excluding repetitive elements, such as genes in the PE/PPE family and insertion elements (S1 Table) which are difficult to confidently describe due to the technological limitations of short-read next-generation sequencing, between 3 and 24 genes were deleted in each strain (S2 Table). For the most part, these deletions corresponded to the previously described regions of difference [28]. We also identified large-scale duplications present in a subset of the clinical strains, encompassing up to 309 genes (S3 Table). Similar duplications have previously been observed in Mtb strains and appear to be the result of unequal homologous recombination mediated by IS6110 elements [29–31]. In addition to structural variants, we identified genes disrupted by nonsense and frameshift mutations and insertion elements (S4 Table). Several hundred SNPs between each strain and the reference genome, including non-synonymous polymorphisms in the coding regions of a number of genes, were also identified (S5 Table).

Transposon-junction reads were then mapped to each strain’s genome assembly (Fig 1B), revealing that the Mtb clinical strains were amenable to transposon mutagenesis, with ~50 to 70% of *Himar1* transposon insertion sites (TA dinucleotides) in each library containing one or more insertions (S6 Table). This is comparable to the saturation of the H37Rv libraries generated in this study and previously published Mtb transposon libraries [17,19]. Library reproducibility was also high, as determined by comparing transposon insertion count across each gene between library replicates (Spearman correlation coefficient = 0.81–0.99, S6 Table). We then sought to develop a rigorous analytic approach to compare transposon-junction sequencing data across these genetically diverse strains in order to identify differences in their genetic requirements for *in vitro* growth.

### TnSeq reveals differences in the genetic requirements for *in vitro* growth among Mtb clinical strains

TnSeq is used to identify essential genes under a given growth condition, defined as genes unable to tolerate transposon insertions due to the severe fitness cost of disruption. Doing so involves statistical analyses of transposon insertion location and frequency across a coding region, ultimately making a qualitative classification (essential *v*. non-essential) from quantitative insertion count data. Yet performing discrete comparisons of essential genes between strains or conditions overlooks quantitative differences in transposon count between genes with the same qualitative classification. For example, a gene able to tolerate transposon insertions in two strains could be classified as non-essential in both strains, even if the relative abundance of these transposon mutants in their respective library pools differs significantly. Such differences in transposon mutant abundance reflect differences in the fitness cost of gene disruption in a particular genetic background, and indicate the relative dependency of a strain on the gene for growth and survival [16,32,33]. We sought to identify genes with such differential requirements among our panel of strains, incorporating the sequence variants identified through our WGS pipeline.

To identify differences in the genetic requirements for growth among strains, we used a permutation test-based method to identify genes with statistically significant differences in transposon insertion count between strains [34,35]. Briefly, this method calculates the difference in transposon insertion count across a gene between two strains and compares this value to a null distribution of differences generated by randomly reshuffling the insertion counts among the data sets being compared, identifying genes that have a difference in insertion count more extreme than would be expected by chance. To account for the sequence variants identified through WGS and permit comparative analyses between strains, we placed the TA dinucleotides onto a common indexing system based on coordinates in H37Rv. Deleted genes, genes in the duplicated region, and repetitive elements, such as PE/PPE/PGRS genes and insertion elements, were excluded from subsequent analyses (S1 Fig).

**Fig 2:**
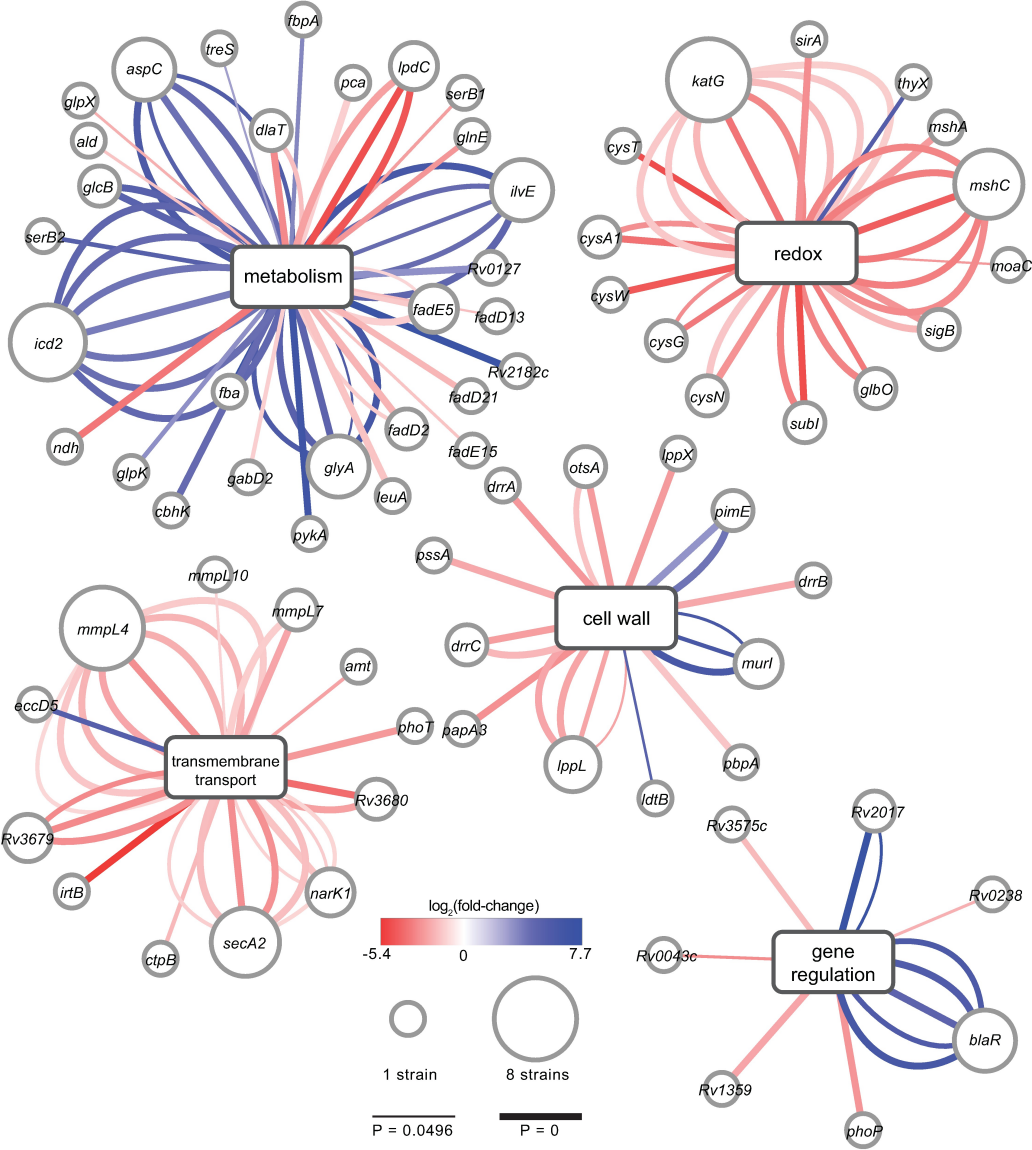
Comprehensive identification of differential genetic requirements for *in vitro* growth by TnSeq. Graphic representation of selected genes with differential requirements for *in vitro* growth across 8 Mtb clinical strains. Diagram was generated using Cytoscape [60]; gene nodes are connected to central nodes representing a functional category; functional category was assigned based on Gene Ontology classification [61,62] and review of the literature. The size of each gene node scales with the number of clinical strains with a statistically significant change in transposon insertion count across that gene as compared to H37Rv (Benjamini-Hochberg adjusted P < 0.05, fold-change ≥ 2 or ≤-2). Each edge represents a strain with a significant difference in transposon insertion count; edge width is inversely scaled to the adjusted P-value as indicated in the legend, and edge color is scaled to the log_2_ fold-change values as indicated by the color scale.

**Fig 3:**
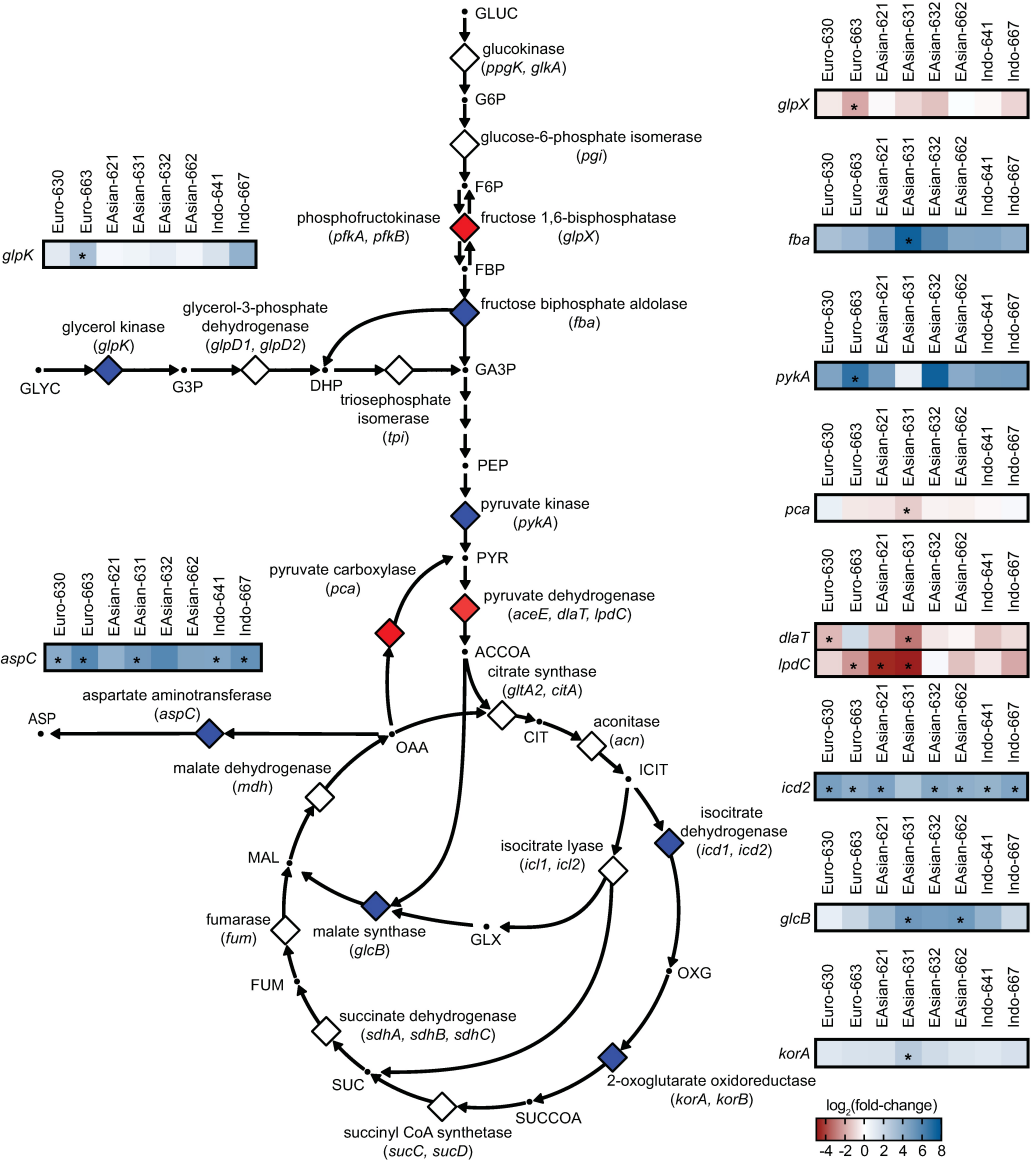
TnSeq reveals differences among strains in requirements for genes in central carbon metabolism pathways. Schematic of central carbon metabolism in Mtb highlighting genes with statistically significant differences in transposon insertion count between clinical strains and the reference strain H37Rv. Metabolites are represented by dots; enzymes are represented by diamonds and gene abbreviations are shown next to the reactions they catalyze. Diamonds with blue fill indicates at least one clinical strain has significantly more transposon insertions across the corresponding gene(s) as compared to H37Rv; red fill indicates at least one clinical strain has significantly fewer transposon insertions across the gene(s) as compared to H37Rv. Heatmaps show log_2_ fold-change in transposon insertion count for this set of genes across all strains as indicated by the color scale; asterisk indicates statistical significance (Benjamini-Hochberg adjusted P < 0.05, fold-change ≥ 2 or ≤-2). Metabolite abbreviations: ACCOA, acetyl-CoA; ASP, aspartate; CIT, citrate; DHP, dihydroxyacetone phosphate; F6P, fructose-6-phosphate; FBP, fructose 1,6-bisphosphate; FUM, fumarate; G3P, glycerol 3-phosphate; G6P, glucose-6-phosphate; GA3P, glyceraldehyde 3-phosphate; GLUC, glucose; GLX, glyoxylate; GLYC, glycerol; ICIT, isocitrate/citrate; MAL, malate; OAA, oxaloacetate; OXG, 2-oxoglutarate; PEP, phosphoenolpyruvate; PYR, pyruvate; SUC, succinate; SUCCOA, succinyl-CoA. The metabolic pathway schematic was generated with Omix [63].

Pairwise comparison of each clinical strain’s transposon libraries to the H37Rv control libraries identified between ~10 and ~50 genes with a statistically significant difference in transposon insertion count per strain (adjusted P-value <0.05 and magnitude fold-change ≥ 2) (S7 Table). Many of the genes with differential genetic requirements are involved in fundamental cellular processes (Fig 2). A number are involved in redox homeostasis, including the catalase-peroxidase (*katG*, increased genetic requirement in all clinical strains compared to H37Rv), genes in the mycothiol synthesis pathway (*mshA*, increased requirement in East Asian 631; *mshC*, increased requirement in Euro-American 630, 663, East Asian 621, 631, Indo-Oceanic 641, 667), and genes involved in sulfur uptake and assimilation (*cysW*, *cysT*, *subI*, *cysN*, *cysA1*, increased requirement in Euro-American 630, 663), processes that support synthesis of mycothiol and other biomolecules involved in redox homeostasis such as thioredoxins (Fig 2, S7 Table). There were also numerous differences among strains in the requirements for genes whose products mediate central metabolism (Fig 3, S7 Table). The genes in this group included genes in the pyruvate dehydrogenase complex (*dlaT*, increased genetic requirement in Euro-American 630, East Asian 631; *lpdC*, increased requirement in Euro-American 663, East Asian 621, 631) and malate synthase (*glcB*, decreased genetic requirement in East Asian 621, 662). Some of the enzymes encoded by these genes are key regulators of entry into the tricarboxylic acid (TCA) cycle (*dlaT*, *lpdC*), and may indicate differences in metabolic flux in these strains.

### Differential genetic requirements are not predicted by sequence variants or gene expression

Our results demonstrate that TnSeq is a scalable approach to identify functional genetic differences among Mtb strains. Yet TnSeq is more technically challenging than WGS or gene expression methodologies. Therefore, we sought to determine whether the differences in genetic requirements identified by TnSeq could have been predicted from WGS or expression data.

**Fig 4:**
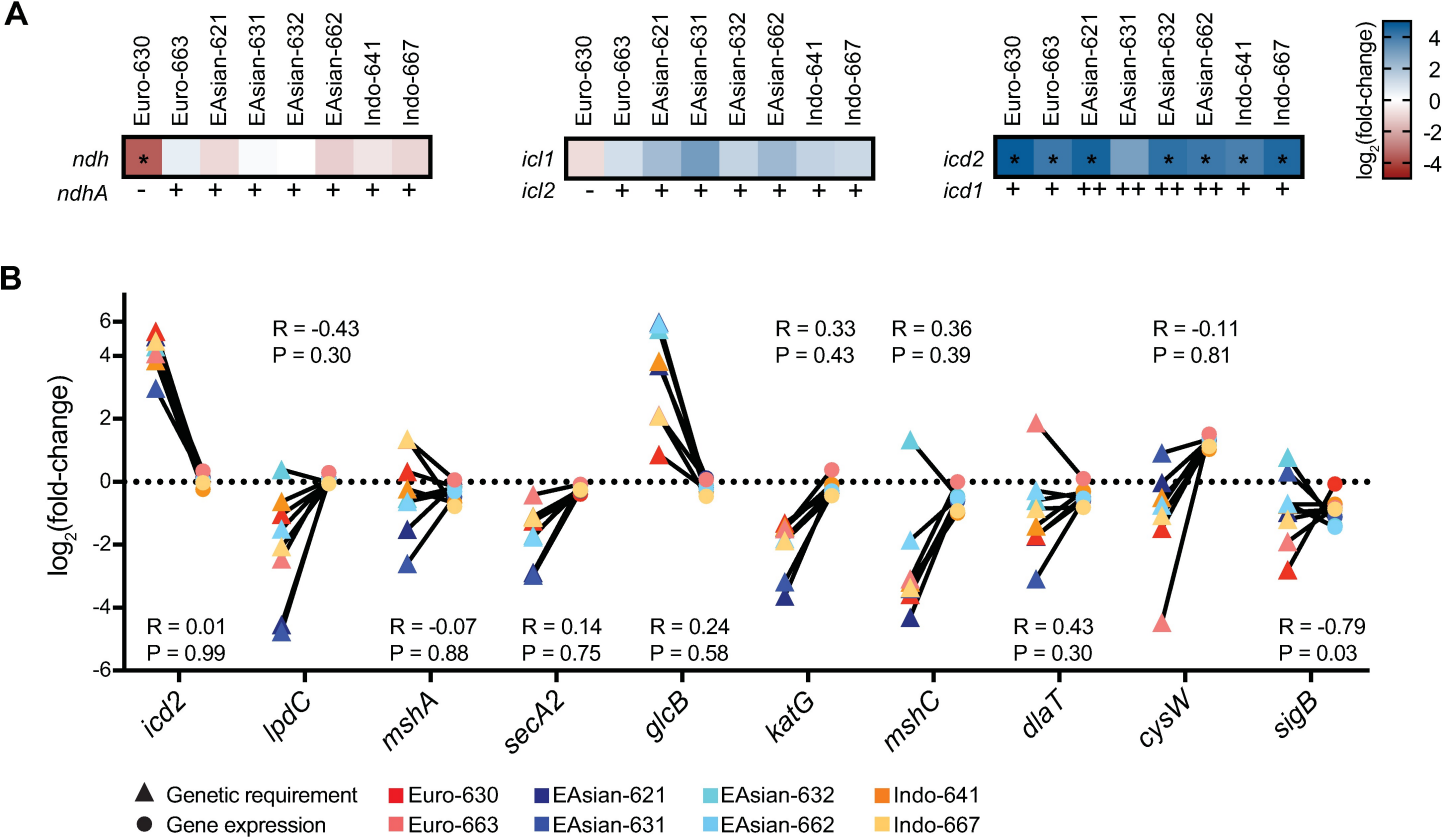
Strain-specific genetic requirements are not predicted by sequence variants or gene expression. (A) Genetic variants identified by WGS generally do not correspond to differences in genetic requirements. Heatmaps show log_2_ fold-change in transposon insertion count across *ndh*, *icl1*, or *icd2* relative to H37Rv as indicated by the color scale; asterisks indicate a statistically significant difference (Benjamini-Hochberg adjusted P < 0.05, fold-change ≥ 2 or ≤-2). Below the heatmap, an intact open reading frame for *ndhA* or *icl2* is indicated by “+” while a disrupted open reading frame is indicated by “-”; strains with a duplication involving *icd1* are indicated by ‘++’ while no duplication is indicated by ‘+’. (B) Correlation between genetic requirements predicted by TnSeq and gene expression. Y-axis is log_2_ fold-change in transposon insertion count relative to H37Rv (triangles) or mean log_2_ fold-change in gene expression relative to H37Rv as measured by NanoString assay (circles). Spearman rank correlation coefficient between log_2_ fold change in transposon count and expression and associated P-values are shown for each gene.

We first assessed whether any of the differential genetic requirements could have been predicted by sequence variants identified through our WGS pipeline. A differential genetic requirement could be attributed to a specific polymorphism in only one case. Euro-American strain 630 had an ~12-fold decrease in transposon insertion count across *ndh*, the type II NADH dehydrogenase, relative to H37Rv, indicating an increased genetic requirement for this gene (Fig 4A). Our WGS pipeline revealed that strain 630 has a nonsense mutation in *ndhA*, a paralog of *ndh* [36], suggesting that a loss of functional redundancy created a relative requirement for the remaining NADH dehydrogenase (Fig 4A, S4 Table). In another instance of apparent loss of redundancy, *icl2*, encoding one of two isocitrate lyase enzymes in the TCA cycle [37], is disrupted by a frameshift mutation in the reference strain H37Rv and Euro-American strain 630, but is intact in the other clinical strains in our panel (S4 Table). In the strains with an intact *icl2*, there was a non-significant increase in transposon insertion count across the paralog, *icl1*, compared to H37Rv (Fig 4A).

In many other instances the genetic basis for differences in genetic requirements between strains was not clear. For example, our WGS pipeline identified large-scale duplications in a subset of the clinical strains encompassing the TCA cycle gene *icd1*, which encodes one of two isocitrate dehydrogenases (S3 Table) [38]. The functional redundancy created by this duplication might decrease the genetic requirement for the other isocitrate dehydrogenase isoform, encoded by *icd2*, for strains with two copies of *icd1*. However, we observed a decreased genetic requirement for *icd2* across all clinical strains, even those without the duplication (Fig 4A). Thus, in most cases the relationship between genetic requirements and sequence variants are not readily apparent, likely reflecting the complexity of genetic networks.

Transcriptomic studies have identified differences in gene expression between Mtb clinical strains [39,40], therefore, we next assessed whether genes with differential requirements for growth display corresponding differences in expression. We selected a set of 10 genes identified as differentially required between strains by TnSeq, prioritizing genes of known function, and determined expression by NanoString (see Materials and Methods). For all but one gene, the sigma factor *sigB*, there was no correlation between transposon insertion count across a gene and gene expression relative to the reference strain H37Rv (Fig 4B). These findings suggest that differences in genetic requirements between strains cannot be predicted by expression data, consistent with comparisons of gene expression and gene essentiality within a strain [41].

### Genetic requirement for *katG* predicts isoniazid resistance rate

We then sought to validate some of the functional differences among strains predicted by our TnSeq data. Because strain diversity has the potential to impact the efficacy of drugs, we focused on genes whose products are the targets of antibiotics. Our TnSeq data indicated that all of the clinical strains have an increased requirement for several genes implicated in redox homeostasis including the gene encoding the catalase-peroxidase, KatG, as compared to H37Rv (Fig 2, S7 Table). KatG is the activator of isoniazid (INH), a key first-line anti-tubercular agent that is converted to its active form by the bacterial catalase-peroxidase in a redox sensitive fashion. We sought to determine whether the differences in genetic requirement for *katG* would predict differences in response to INH. We first determined minimum inhibitory concentration (MIC) for H37Rv and the panel of clinical strains (see Materials and Methods). The MICs for all strains were within 2-fold that of H37Rv, with the exception of Indo-Oceanic strain 667, which had an MIC 4-fold lower than H37Rv (S8 Table). Thus, the differential genetic requirement for *katG* did not correspond to differences in INH susceptibility.

A major mechanism of acquired resistance to INH is through *katG* mutation. In laboratory studies, INH resistance occurs at a high rate, primarily through mutations that result in a loss of KatG function, including complete and partial deletions of the gene [42,43]. However, in clinical studies, a relatively small number of *katG* mutations account for most INH resistance. A single SNP (S315T), which preserves some catalase-peroxidase activity, accounts for over two-thirds of INH resistance in some studies, and *katG* deletions are rare [44,45]. The increased genetic requirement for *katG* among clinical strains identified by TnSeq might explain these observations.

**Fig 5:**
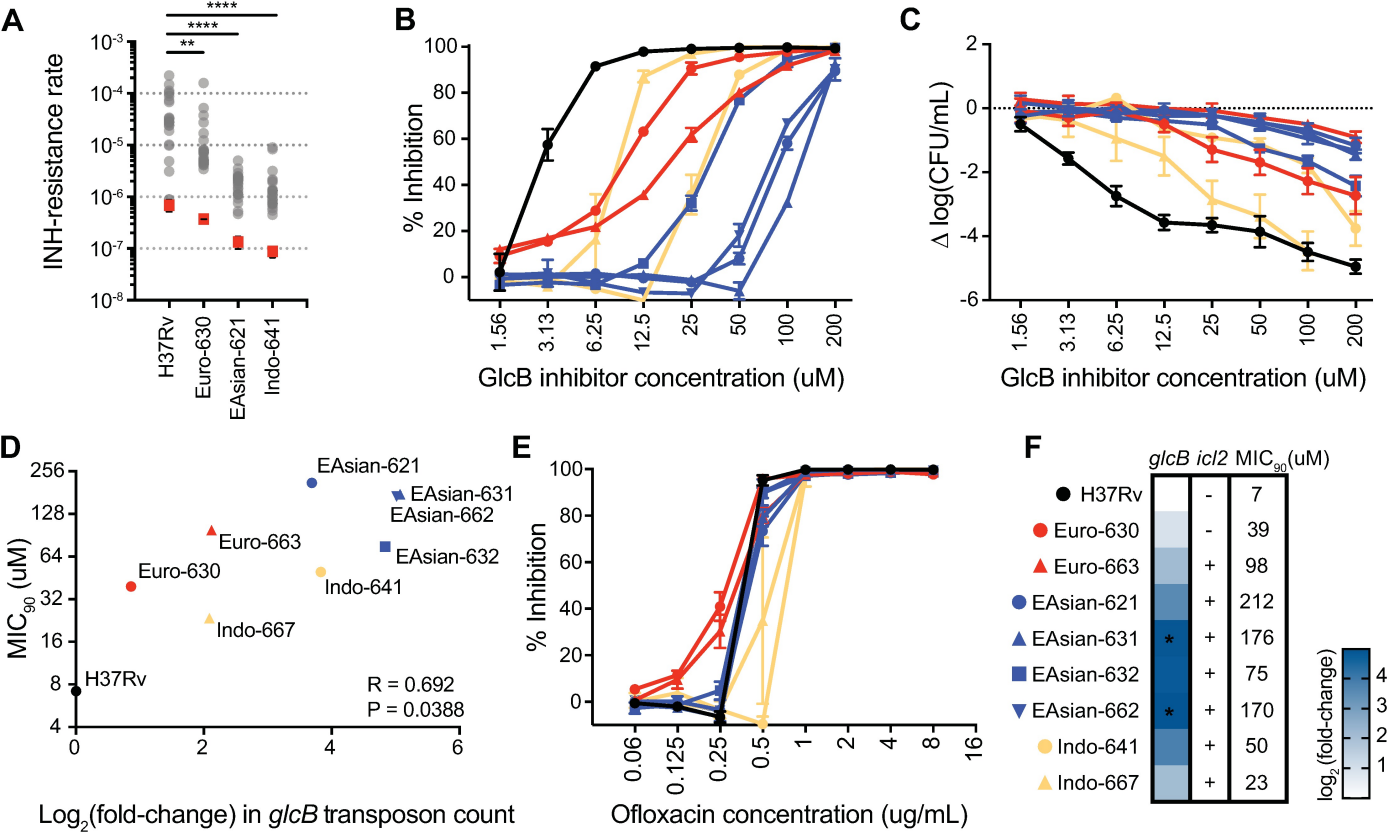
Differential genetic requirements identified by TnSeq predict antibiotic phenotypes. (A) Fluctuation analysis was used to measure the rates at which clinical strains and the reference strain H37Rv acquired resistance to isoniazid (1 μg/mL). Circles represent mutant frequency (number of resistant mutants per cell plated for a single culture). Red squares represent the estimated mutation rates, calculated as described in [3], with error bars representing the 95% confidence intervals. Significant differences in mutation rate were defined by non-overlapping 95% confidence intervals and significance was determined by comparing each clinical strain’s mutation rate to the H37Rv mutation rate using the Wilcoxon rank-sum test (Mann-Whitney *U* test); ** P < 0.005, **** P < 0.0001. (B) The microplate Alamar Blue assay was used to determine MIC_90_ for H37Rv and 8 clinical strains for a novel small-molecule GlcB inhibitor. Shown is the normalized inhibition of Alamar Blue conversion at each concentration from a representative experiment. (C) Change in CFU/mL for each strain after incubating for 8 days at the indicated concentration of GlcB inhibitor. (D) Plot of MIC_90_ *v*. log_2_ fold-change in transposon insertion count across the *glcB* gene relative to H37Rv. (E) Strains do not differ in their susceptibility to ofloxacin. Normalized inhibition of Alamar Blue conversion at each concentration of ofloxacin from a representative experiment. (F) Genetic variants in the glyoxylate shunt do not predict susceptibility to the GlcB inhibitor. For each strain, the log_2_ fold-change in transposon insertion count across *glcB* relative to H37Rv is shown in the heatmap; to the right of the heatmap, an intact *icl2* open reading frame is indicated by “+”, while a disrupted open reading frame is indicated by “-”; to the far right, MIC_90_ for the GlcB inhibitor is indicated. Color and symbol to the left of each strain is maintained in (B-F).

We reasoned that the increased genetic requirement for *katG* among clinical isolates might reduce the number of viable mutations that confer INH resistance (target size), resulting in a lower INH resistance rate [42]. To test this hypothesis, we measured the rate at which INH resistance is acquired by performing Luria-Delbruck fluctuation analysis on three clinical strains from our panel, one from each lineage, and H37Rv. Consistent with the prediction from our TnSeq data, all three clinical strains acquired resistance to INH (1 μg/mL) at a significantly lower rate than H37Rv (Fig 5A). Lineage-based differences in basal mutation rate that impact the likelihood of drug resistance have previously been described [3], and additional work has found sub-lineage and strain-specific differences in mutation rate (personal communication, Sarah Fortune and Nathan Hicks). Therefore, to confirm that the decreased rate of resistance to INH among the clinical strains was not due to a lower basal mutation rate, we also determined the rate of resistance to an antibiotic that targets a different cellular process, rifampicin. The rifampicin resistance rates were not different between H37Rv and any of the clinical strains tested (S2 Fig). Together, these findings suggest that the fitness costs of disrupting genes that encode antibiotic activators or targets can also contribute to the frequency of emergence of drug resistance, consistent with findings from population-based studies [6,9].

### Genetic requirement for *glcB* predicts susceptibility to a small-molecule inhibitor of malate synthase

We identified a number of central carbon metabolism genes with differential genetic requirements from our TnSeq data (Fig 3). Bacterial metabolism has emerged as a promising target for antibiotic development [46,47] but our data suggests that molecules targeting these pathways may have variable efficacy across clinical strains. For example, the clinical strains varied in their requirement for *glcB*, which encodes malate synthase (GlcB), the second enzyme in the glyoxylate shunt (Fig 3, S7 Table). The glyoxylate shunt is an anaplerotic pathway important for central carbon and fatty acid metabolism and the enzymes in this pathway, malate synthase and isocitrate lyase, play multiple, essential roles in establishing and maintaining *in vivo* infection [48–51]. It is also a pathway present only in prokaryotes, plants, and fungi. These features make the glyoxylate shunt an attractive drug target.

Indeed, a novel GlcB inhibitor has recently been developed that is effective in reducing the burden of H37Rv in a mouse model of infection [52]. This inhibitor was developed through a structure-guided optimization of phenyl-diketo acids [52]. The inhibitors of this series bind to the active site of GlcB by coordinating the catalytic Mg^2+^ ion with the ketoacid moiety, making hydrogen bonds with the catalytic acid, arginine, through both keto groups, and engaging in anion-π interactions with the catalytic base, asparagine, through the phenyl group. These amino acid moieties are conserved at the sequence level across the clinical strains (the only SNPs in *glcB* are G104S in the four East Asian strains, and T191A in Indo-Oceanic strain 641, S5 Table), but our TnSeq data suggested that the genetic requirement for *glcB* varied between strains, potentially altering sensitivity to the inhibitor.

We hypothesized that strains with a decreased genetic requirement for *glcB* during *in vitro* growth would be less sensitive to GlcB inhibition in culture. To test this hypothesis, we used the 2-Cl-6-F-3-Me-Phenyl diketo acid member of the inhibitor series, which was developed for the favorable pharmacological qualities needed to chemically evaluate GlcB as a target in Mtb infection [52]. The acid form inhibits purified GlcB with an IC_50_ of 5.5 μM, and the esterified form, which has improved cellular uptake, kills H37Rv grown on acetate with an MIC_99_ of 2 μM and kills H37Rv grown on dextrose in the presence of fatty acids with an MIC_99_ of 8 μM.

We determined MIC for the esterified form of the GlcB inhibitor by Alamar Blue assay for bacteria grown on acetate with dextrose (see Materials and Methods) for all strains (Fig 5B). Strains differed in their MIC_90_ up to 30-fold, from 7 μM (H37Rv) to 212 μM (East Asian 621). The strains with a significant increase in transposon insertion count across *glcB* (East Asian 631, 662) had MIC_90_s ~25- and ~24-fold higher than H37Rv, respectively. The increase in MIC_90_ corresponded with reduced bacterial killing (Fig 5C). Across all strains, the log_2_ fold-change in transposon insertion count across *glcB* correlated with MIC_90_ (R = 0.692, P = 0.0388) (Fig 5D). We also determined the MICs for ofloxacin and streptomycin, which did not differ by more than 2-fold among strains (Fig 5E, S3 Fig, S8 Table). Thus, the differences in sensitivity to the GlcB inhibitor are specific to this pathway and do not reflect general differences in drug susceptibility among strains.

Finally, we assessed whether differences in sensitivity to the GlcB inhibitor could have been predicted by genetic variants identified through WGS or by gene expression. The first step in the glyoxylate shunt, hydrolysis of isocitrate into glyoxylate and succinate, is catalyzed by isocitrate lyases encoded by two paralogous genes: *icl1* and *icl2* (Fig 3). As described above, *icl2* is disrupted by a frameshift mutation in H37Rv, yet many strains retain an intact open reading frame and express a second, functional isocitrate lyase [37]. The *icl2* frameshift was present in only one of our clinical strains (Euro-American strain 630) while the other strains in the panel possessed an intact *icl2* (Fig 5F, S4 Table). Thus, *icl2* status could not be used to predict susceptibility to the GlcB inhibitor among these strains. Expression of *glcB* was not significantly different between H37Rv and any of the clinical strains (Fig 4B, ANOVA with Tukey’s post test). Taken together, these results indicate that our TnSeq data can predict susceptibility to a novel antibiotic that could not be readily derived from sequence or expression data alone.

## Discussion

Despite the relatively conserved genomic content of Mtb, strains can vary dramatically in features that impact clinical outcomes, including capacity to acquire antibiotic resistance [3,6]. To investigate the basis of strain variation, we used TnSeq to define differences in the genetic requirements for *in vitro* growth across a panel of 9 genetically diverse Mtb strains. To date, this represents the largest comparative TnSeq study of a microorganism and provides an analytic framework for multi-strain TnSeq comparisons in bacteria.

We identified as many as ~50 differentially required genes in each strain relative to the reference strain, H37Rv, for *in vitro* growth. These included differences in the requirements for genes involved in fundamental cellular processes, such as central carbon metabolism. Some of these differences occurred in all strains in the panel, perhaps reflecting adaptation of the reference strain to laboratory culture. These findings underscore the potential pitfalls of relying on reference strains to infer the common biology of a species. Other genes were differentially required in only a subset of strains or were strain-specific. Mtb has a geographically determined phylogenetic structure that divides into seven major lineages, yet we did not find that differences in genetic requirements tracked strictly with lineage. While it is tempting to think of Mtb strains within a lineage as having collective behavior, our data is consistent with other studies showing substantial genomic and phenotypic diversity within lineages [24,53].

As with any screening technique, not all biologically important differences can be captured by TnSeq. Two limitations of TnSeq are polar effects and the possibility for *trans* complementation. Polar effects, whereby transposon insertion disrupts not only the function of the gene containing the insertion, but also downstream genes in an operon, are mitigated by the design of the transposon, which contains a strong, outward facing promoter [54]. An earlier study found no insertion bias toward the 5’ end of operons, suggesting polar effects are negligible in this system. Complementation in *trans*, whereby a transposon mutant’s phenotype is suppressed by bacteria wild-type for the locus present in the transposon library, has been reported in some TnSeq studies [55]. Such effects are expected to be limited to secreted products, and could be identified in future studies by phenotypic screening of arrayed transposon libraries.

A key advantage of TnSeq is that it uses the cell as a sensor to directly report on the genes and pathways required for microbial growth. This stands in contrast to WGS, where the selective pressures generating sequence variants may not be known. This feature of TnSeq allowed us to use differences in genetic requirements between strains to predict differences in antibiotic responses. Because many antibiotics target processes essential for growth, we reasoned that a change in genetic requirement for an antibiotic target could predict differences in antibiotic sensitivity. Our *glcB* data validate this approach, which could be used to prioritize targets for drug development, since antibiotics should ideally be effective against all strains of a pathogen. Another important consideration in antibiotic development is the likelihood that resistance will emerge [56]. Some mechanisms of antibiotic resistance, such as the acquisition of inactivating enzymes, activation of efflux pumps, or decrease in antibiotic uptake, may not be detected by TnSeq. Yet our *katG* data indicate that the likelihood of loss of function mutations in antibiotic activators, an important resistance mechanism, can be predicted by TnSeq.

Our findings that TnSeq identifies biologically important differences among strains that are not readily predicted by WGS data highlights a challenge for the field. We are rapidly developing catalogues of bacterial sequence variants but largely lack data to link genotypes and phenotypes. For Mtb, such analyses are confounded by this pathogen’s clonal population structure. Studies in which Mtb genotype has been successfully linked to antibiotic phenotype have required WGS data from hundreds of strains [6,8,11–13]. With TnSeq, we were able to use a much smaller set of strains to predict antibiotic phenotypes. Yet our analyses suggest that attributing differences in genetic requirements to specific sequence variants will require much larger data sets to achieve the statistical power to dissect such interactions.

Here we demonstrate that TnSeq is a scalable method to help close the gap between genotype and phenotype. Importantly, TnSeq can be used to predict strain-specific phenotypes that are not readily predicted from sequence or expression data alone. Given the potential consequences of strain variation for the efficacy of antibiotics and vaccines, this approach could be utilized under other *in vitro* and *in vivo* conditions to assess the generalizability of new therapeutic approaches. Moving forward, integrating TnSeq data with orthogonal approaches, such as proteomics, metabolomics, and transcriptomics, will doubtless provide further insight into the basis of Mtb’s phenotypic heterogeneity. This work provides a foundation for these future studies and highlights the cellular pathways that may be subject to the most variation in Mtb.

## Materials and methods

### Bacterial strains

Clinical strains were identified as previously described and cultured from single colonies [25]. Strains were grown at 37°C and cultured in Middlebrook 7H9 salts supplemented with 10% OADC, 0.5% glycerol and 0.05% Tween-80 or plated on 7H10 agar supplemented with 10% OADC, 0.5% glycerol and 0.05% Tween-80 unless otherwise noted. Clinical strains were handled to minimize *in vitro* passaging.

### Whole genome sequencing

Genomic DNA was isolated from a 10-mL culture of each clinical strain using standard phenol-chloroform techniques. Libraries were prepared with the Illumina Nextera XT kit and sequenced on an Illumina MiSeq Desktop Sequencer with MiSeq Reagent Kit v2. Paired-end sequencing with read lengths of 151 bases was performed for an average coverage of 150x (range 62-240x).

### Genome assemblies and variant calling

Genome sequences of the clinical strains were assembled with a custom comparative-assembly pipeline [57] using *M*. *tuberculosis* H37Rv (GenBank accession number NC_000962.2) as the reference genome. Contig-building was used to define large-scale insertions and deletions (up to 40kb), as well as to infer changes in genomic locations of IS6110 transposons.

Variants including SNPs, insertions, and deletions, were identified by alignment with the reference sequence, except in repetitive elements, such as transposases and PE/PPE/PE-PGRS genes, due to the technological limitations of Illumina sequencing (S1 Table, S1 Fig). Genes with disrupted open reading frames due to small insertions and deletions and nonsense mutations were identified (S4 Table) as were genes with at least 10% of their open reading frame deleted (S2 Table).

### Transposon mutagenesis

The H37Rv and clinical strain *Himar1* transposon libraries were generated as previously described [26,27]. Libraries were selected for 21 days on 7H10 agar supplemented with 10% OADC, 0.5% glycerol, 0.05% Tween-80 and 0.2% Cas-amino acids (Difco) with 20 μg/mL kanamycin. Two independent libraries of ~100,000 mutants each were generated for each clinical strain and three independent libraries of ~100,000 mutants each were generated for H37Rv.

### Transposon junction sequencing and analysis

Genomic DNA was extracted from each transposon library using standard phenol-chloroform techniques and prepared for transposon-junction sequencing essentially as described [27]. Libraries were sequenced on an Illumina HiSeq. Raw reads were mapped to TA sites in each strain’s genome assembly and read counts were reduced to unique template counts using Transit [35]. Statistical comparisons of insertion counts across open reading frames were performed using a permutation test-based method ("resampling" method in Transit), after placing TA sites on a common indexing system and normalizing for library read-depth [34,35]. Insertions in the central 90% of each gene were considered and a LOESS correction for genome positional bias was performed. P-values were adjusted for multiple comparisons by the Benjamini-Hochberg procedure to control the false-discovery rate at ≤ 5% [35]. Deleted genes, repetitive genes, and genes in the duplicated region were excluded prior to performing comparisons of insertion counts between strains (S1 Table, S2 Table, S3 Table, S1 Fig).

### RNA isolation and gene expression

RNA was isolated from triplicate mid-log cultures for each strain using standard techniques. Briefly, cells from each culture were harvested by centrifugation, resuspended in 1 mL of TRIzol (Thermo Fisher), and disrupted by bead-beating. Chloroform was added to 25% of the total volume and total RNA was isolated using the Direct-zol RNA MiniPrep kit (Zymo Research). Samples were then treated with TURBO DNase (Ambion) for 1 hour to remove residual contaminating genomic DNA, followed by clean-up with RNA Clean & Concentrator columns (Zymo Research). RNA was quantified by Qubit RNA Assay (Thermo Fisher) and 25 ng of RNA was used as input in the NanoString nCounter assay (NanoString Technologies) with custom-designed probes to quantify gene expression. Target sequences are listed in S9 Table. Data were analyzed with nSolver version 4 (NanoString Technologies); raw NanoString counts were normalized to internal positive controls to correct for technical variation between assays, and normalized to a housekeeping gene (*sigA*) to correct for variation in RNA input amount. Background counts from no-input samples were subtracted.

### Fluctuation analysis

Fluctuation analysis and statistical analyses of the data were performed essentially as described previously, with 24 independent cultures per isolate [3].

### Minimum inhibitory concentration determination

Isoniazid MICs were determined by the microplate Alamar Blue assay [58] and the agar proportion method [59]. For the Alamar Blue assay, strains were grown in 7H9 supplemented with OADC, glycerol, and Tween-80 to an OD_600_ of ~1, filtered with a 5 μM filter to obtain a single-cell suspension, then diluted in 7H9 with supplements to an OD_600_ of 0.003 and pipetted in replicate into 96-well plates in the presence or absence of serially diluted isoniazid as described [58]. Plates were incubated at 37°C with shaking for four days before adding Alamar Blue (Bio-Rad), then incubated for an additional two days before visually scoring for color change. MIC was defined as the lowest concentration of antibiotic that prevented color change from blue or purple to pink. For the agar proportion method, 7H10 media supplemented with 10% OADC, 0.5% glycerol and 0.05% Tween-80 was prepared and isoniazid added to attain the following concentrations: 0.8, 0.4, 0.2, 0.1, 0.05, 0.025, and 0.0125 μg/mL. Serial dilutions of duplicate mid-log cultures for each strain were prepared and plated on isoniazid-containing or control plates without antibiotic. Three weeks after plating, CFU were enumerated, and the reported MIC is the isoniazid concentration which reduced CFU by an average of 99% compared to the control.

To determine the MICs for the GlcB inhibitor and control antibiotics streptomycin and ofloxacin, strains were grown in 7H9 supplemented with OADC, glycerol, and Tween-80 to an OD_600_ of ~1, filtered with a 5 μM filter to obtain a single-cell suspension, then diluted into testing media to an OD_600_ of 0.01 and pipetted into 96-well plates in the presence or absence of serially diluted drug as described above. Testing media consisted of 7H9 media supplemented with 0.5% dextrose, 0.1% sodium acetate, 0.085% NaCl, and 0.05% Tyloxapol. Plates were incubated at 37°C with shaking for six days before adding Alamar Blue, then incubated for an additional two days before reading OD_570_ (resorufin) and OD_600_ (resazurin). The difference between these two OD values was used as a viability reporter. The MIC_90_ was derived from curve fit plots generated in Prism and the reported value is the average of two independent experiments. On the day that plates were read, an aliquot from each well was used to make serial dilutions and plate for CFU. MIC experiments were performed in technical replicate at least twice. Plating for CFU was performed in technical replicate once.

## Supporting information

S1 Fig Flowchart illustrating the gene sets included in variant calling and TnSeq analysis. (TIF)

S2 Fig Rifampicin fluctuation analysis. Fluctuation analysis was used to measure the rates at which clinical strains and H37Rv acquired resistance to rifampicin (2 μg/mL). Circles represent mutant frequency. Red squares represent the estimated mutation rates, calculated as described in [3], with error bars representing the 95% confidence intervals. No statistically significant differences in mutation rate, as defined by non-overlapping 95% confidence intervals, were found among these strains.
(TIF)

S3 Fig Streptomycin MICs. Normalized inhibition of Alamar Blue conversion at each concentration of streptomycin from a representative experiment.
(TIF)

S1 Table Repetitive elements. Repetitive genes excluded from variant calling and TnSeq analysis. Annotation is based on the reference genome H37Rv.
(XLSX)

S2 Table Deleted genes. Genes identified through our variant-calling pipeline as having more than 10% of the open reading frame deleted. Genes in repetitive regions (S1 Table) are excluded from this analysis. If the deleted genes correspond to a region of difference, this is noted, with nomenclature according to Tsolaki *et al*.
(XLSX)

S3 Table Description of large-scale duplications. Boundaries of large-scale duplications identified in a subset of the clinical strains. Boundaries were determined by contig-building, that is, a contiguous sequence of overlapping reads spanning the boundary that show the precise nucleotides where an in-del starts or ends.
(TIF)

S4 Table Disrupted genes. Genes with nonsense mutations, small in-dels, or insertion elements that disrupt the open reading frame. Variants in the repetitive gene list (S1 Table) were not called. Note that *icl2* is annotated as *aceAa*/Rv1915 and *aceAb*/Rv1916, consistent with the H37Rv annotation.
(XLSX)

S5 Table Single nucleotide polymorphisms. Coding region SNPs identified in each clinical strain with H37Rv as the reference genome. SNPs in the repetitive gene list (S1 Table) were not called.
(XLSX)

S6 Table Transposon library saturation and correlation between library replicates. Percent of TA dinucleotides with at least one transposon-junction read in each transposon library. Spearman correlation coefficient was determined by comparing the normalized insertion template count across each gene between library replicates.
(TIF)

S7 Table Transit resampling output. N is number of TA sites across the open reading frame; TAs hit is the number of TA sites with at least one insertion; Sum Rd 1 is the normalized sum of unique template read counts in the control (H37Rv) libraries; Sum Rd 2 is the normalized sum of read counts in the clinical strain libraries; Delta Rd is the difference in the normalized sum of read counts; p-value is the P-value calculated by the permutation test; p-adj is the Benjamini-Hochberg adjusted P-value.
(XLSX)

S8 Table Isoniazid, ofloxacin, and streptomycin MICs. MICs determined by Alamar Blue assay or agar proportion method as described in Materials and Methods.
(TIF)

S9 Table NanoString target sequences. Target sequences for gene expression.
(XLSX)
