# Correction: TnSeq of *Mycobacterium tuberculosis* clinical isolates reveals strain-specific antibiotic liabilities

**DOI:** 10.1371/journal.ppat.1007846

**Published:** 2019-06-04

**Authors:** Allison F. Carey, Jeremy M. Rock, Inna V. Krieger, Michael R. Chase, Marta Fernandez-Suarez, Sebastien Gagneux, James C. Sacchettini, Thomas R. Ioerger, Sarah M. Fortune

In the Results section, under the “TnSeq reveals differences in the genetic requirements for *in vitro* growth among Mtb clinical strains” heading, there is an error in the fifth sentence of the third paragraph. The correct sentence is: The genes in this group included genes in the pyruvate dehydrogenase complex (*dlaT*, increased genetic requirement in Euro-American 630, East Asian 631; *lpdC*, increased requirement in Euro-American 663, East Asian 621, 631) and malate synthase (*glcB*, decreased genetic requirement in East Asian 631, 662).
