# Supplementary figures and images for: TnSeq of Mycobacterium tuberculosis clinical isolates reveals strain-specific antibiotic liabilities
Source: PLoS Pathog. 2018 Mar 5;14(3):e1006939. doi: 10.1371/journal.ppat.1006939 (PMC5854444; doi:10.1371/journal.ppat.1006939)

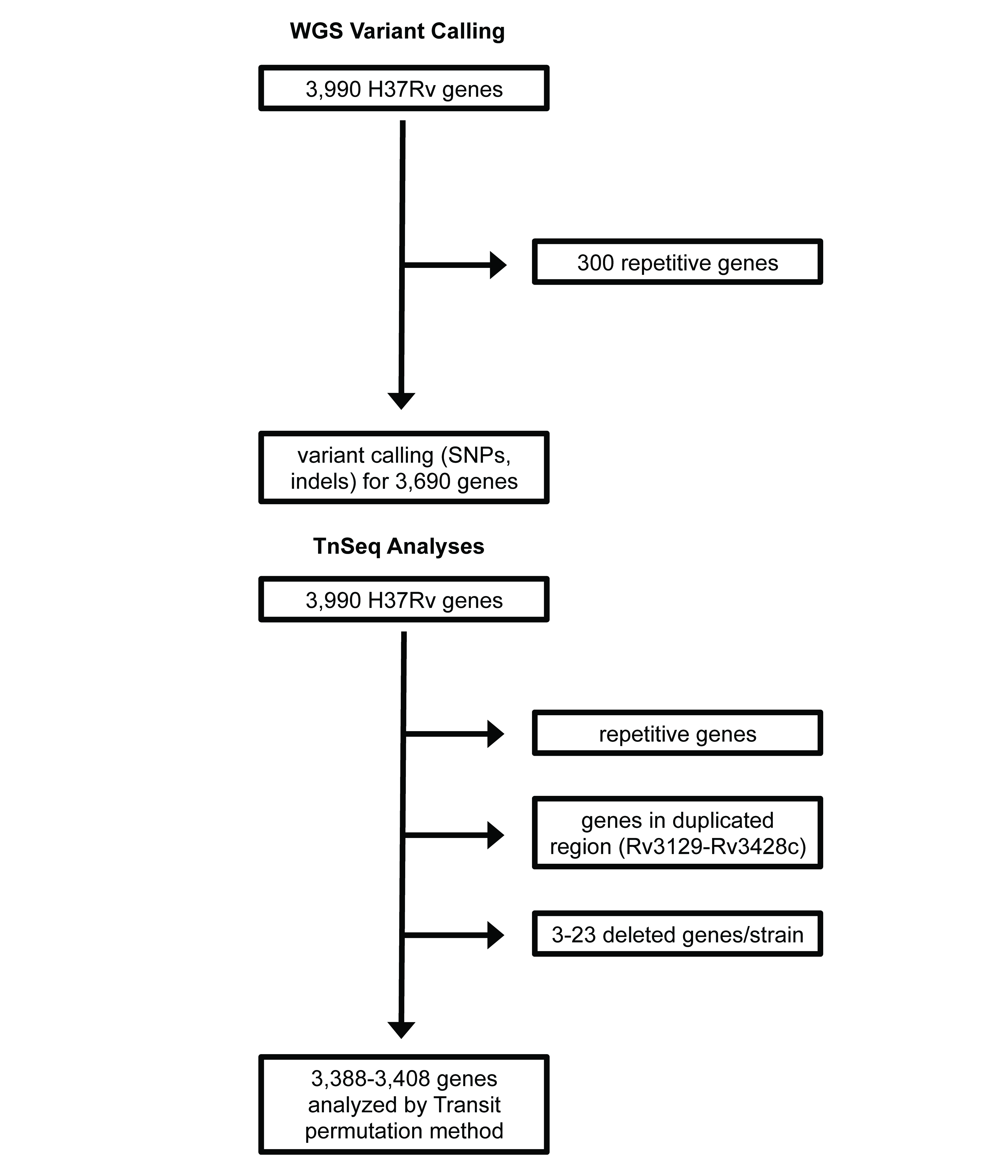

Supplement: S1 Fig — (TIF) [file ppat.1006939.s001.tif]

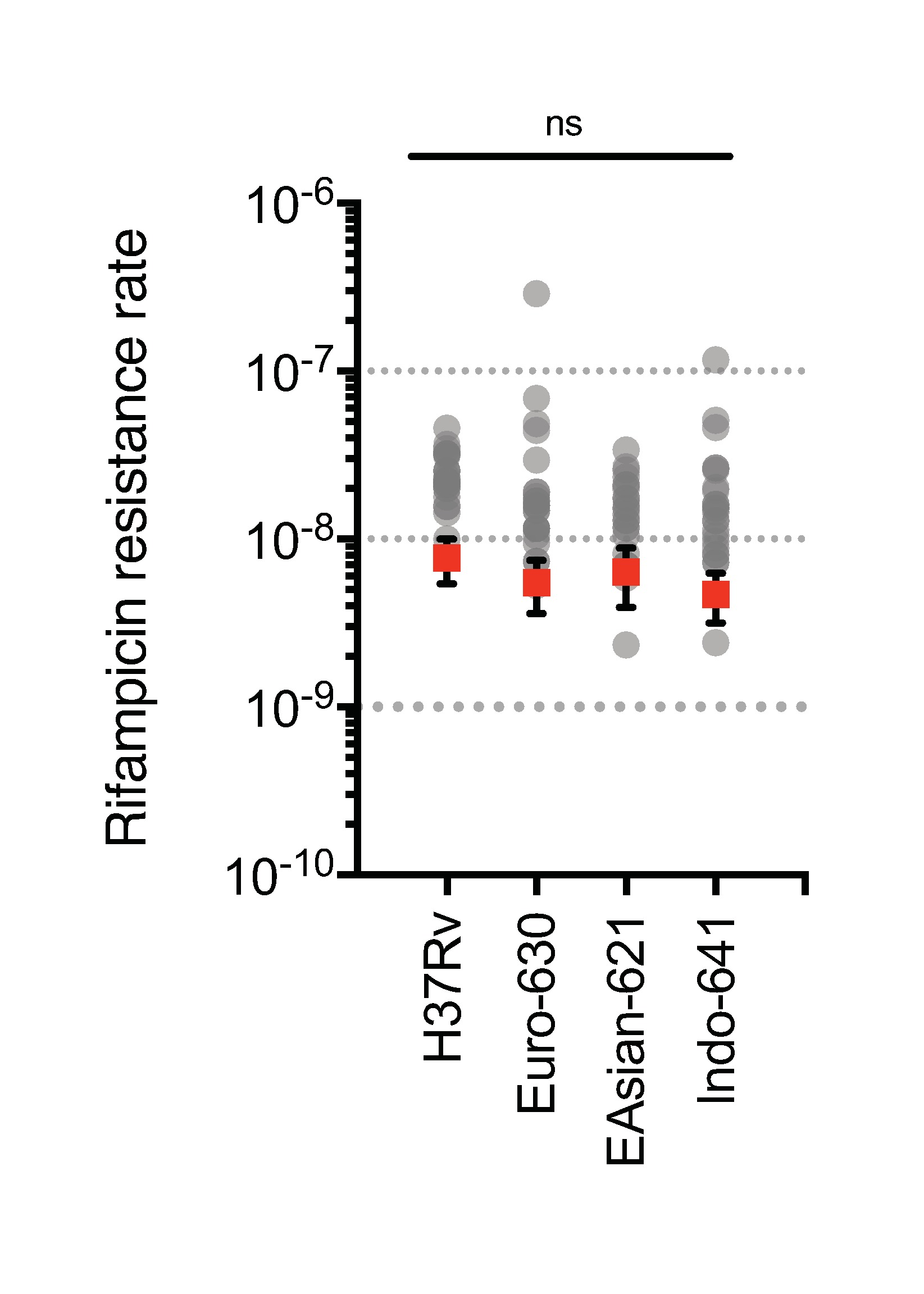

Supplement: S2 Fig — Fluctuation analysis was used to measure the rates at which clinical strains and H37Rv acquired resistance to rifampicin (2 μg/mL). Circles represent mutant frequency. Red squares represent the estimated mutation rates, calculated as described in [3], with error bars representing the 95% confidence intervals. No statistically significant differences in mutation rate, as defined by non-overlapping 95% confidence intervals, were found among these strains. (TIF) [file ppat.1006939.s002.tif]

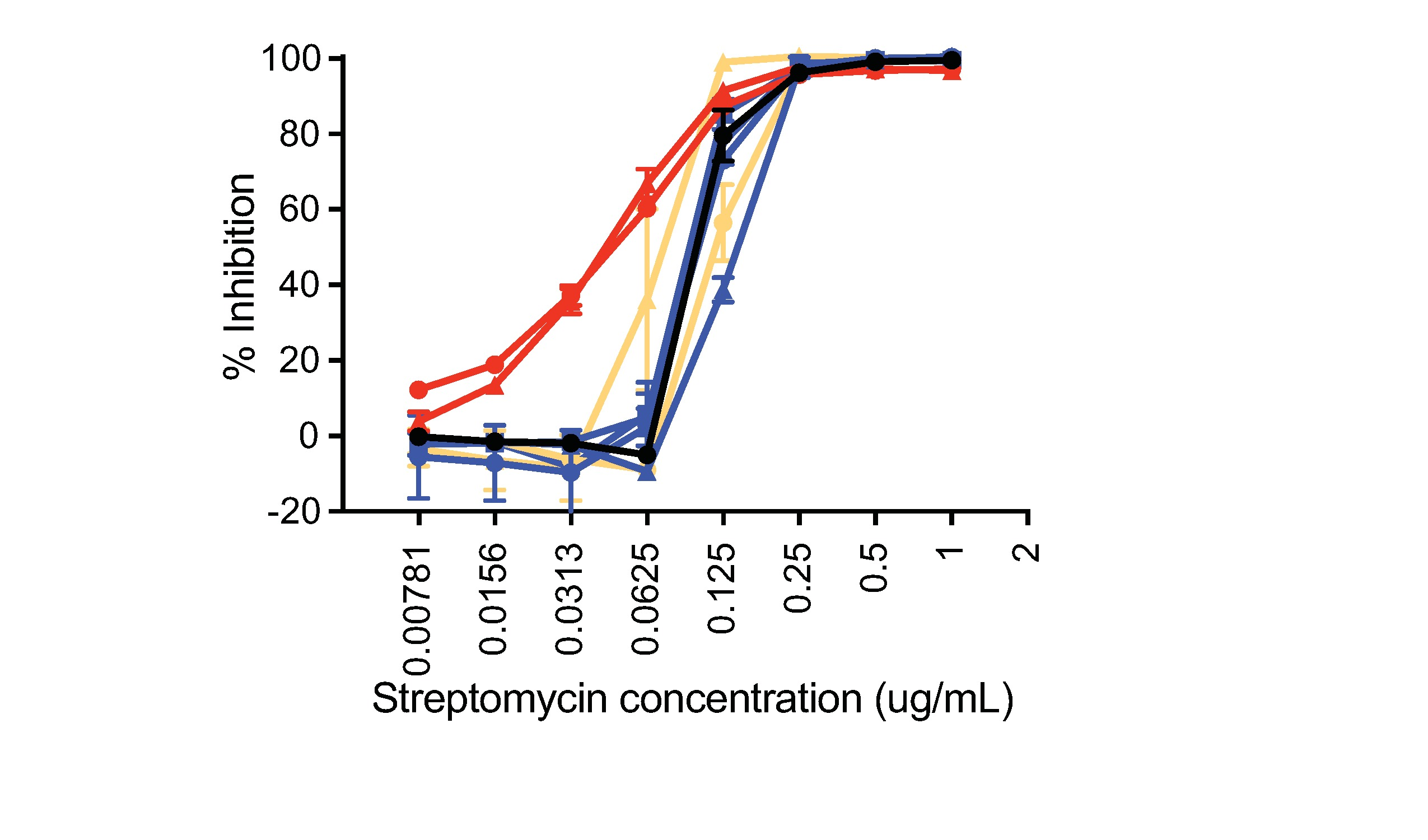

Supplement: S3 Fig — Normalized inhibition of Alamar Blue conversion at each concentration of streptomycin from a representative experiment. (TIF) [file ppat.1006939.s003.tif]

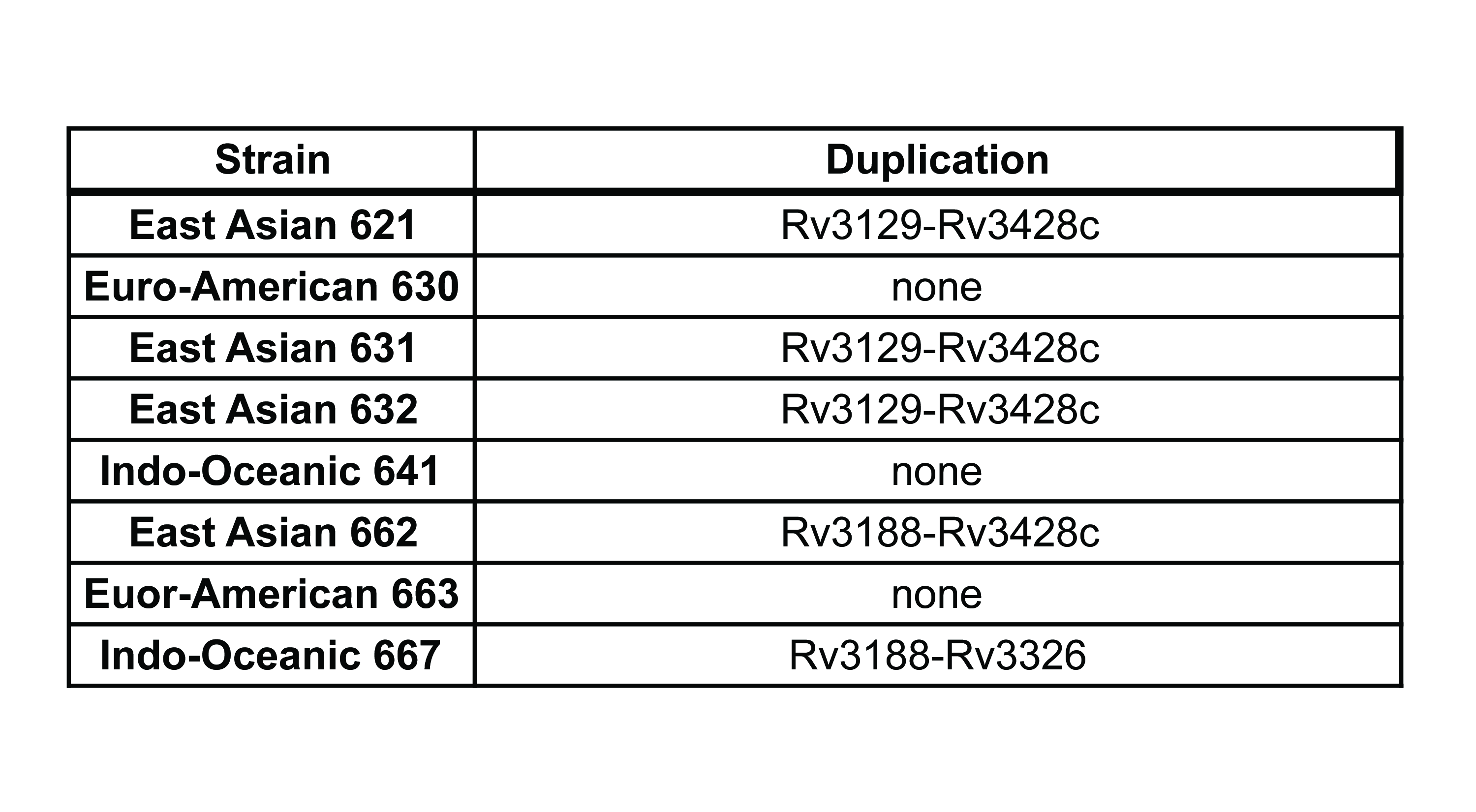

Supplement: S3 Table — Boundaries of large-scale duplications identified in a subset of the clinical strains. Boundaries were determined by contig-building, that is, a contiguous sequence of overlapping reads spanning the boundary that show the precise nucleotides where an in-del starts or ends. (TIF) [file ppat.1006939.s006.tif]

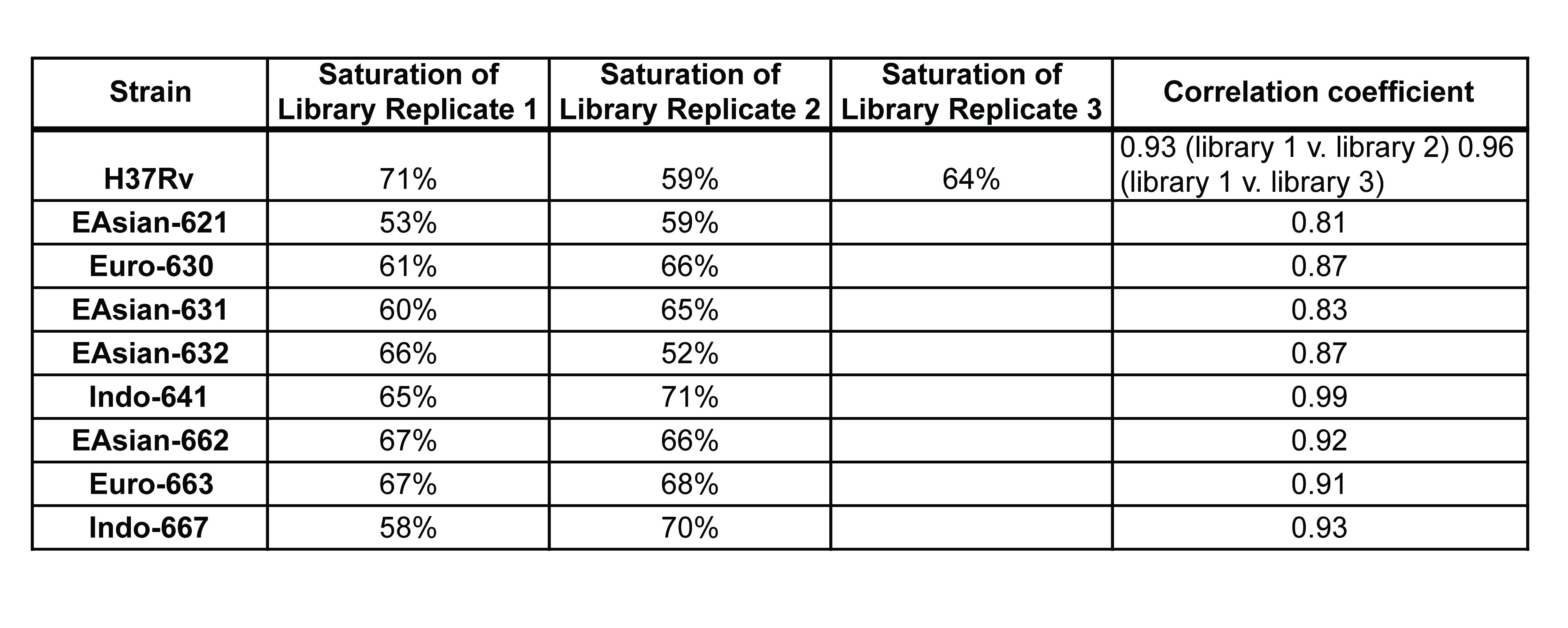

Supplement: S6 Table — Percent of TA dinucleotides with at least one transposon-junction read in each transposon library. Spearman correlation coefficient was determined by comparing the normalized insertion template count across each gene between library replicates. (TIF) [file ppat.1006939.s009.tif]

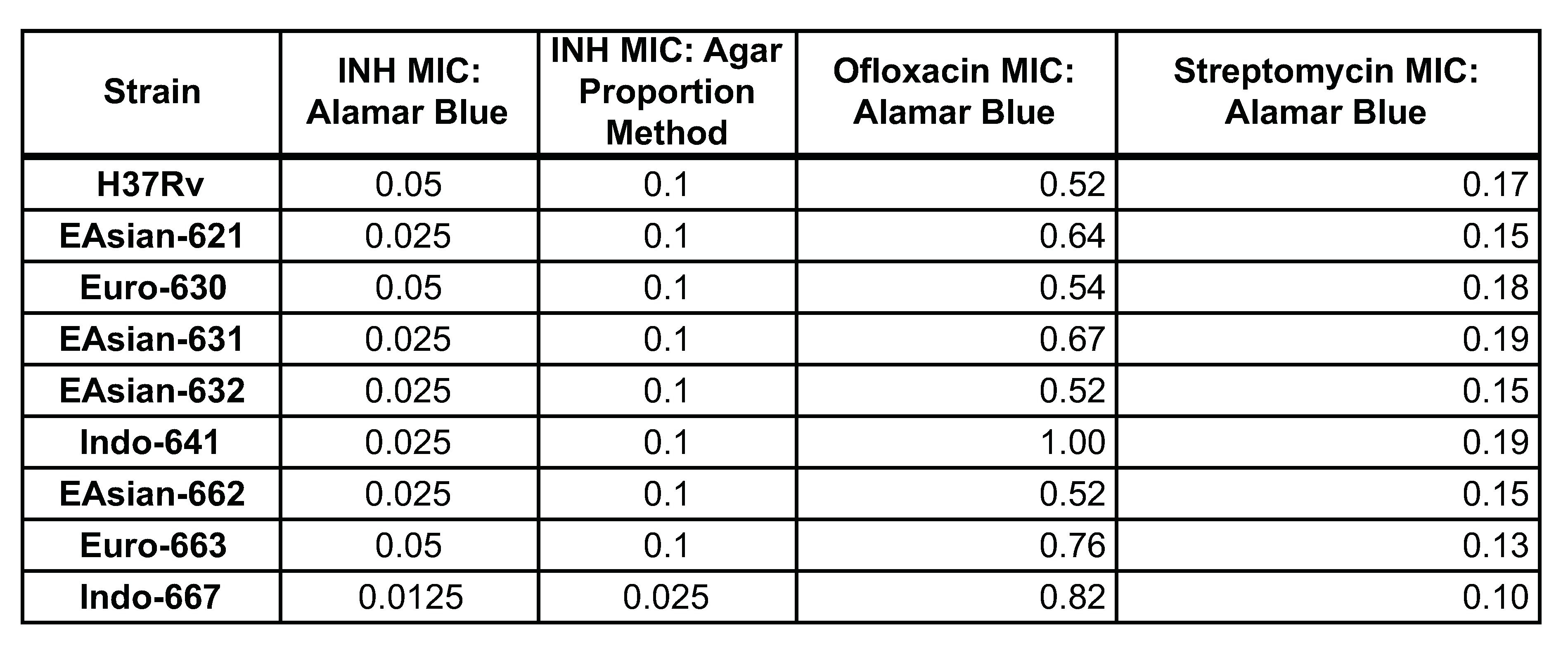

Supplement: S8 Table — MICs determined by Alamar Blue assay or agar proportion method as described in Materials and Methods. (TIF) [file ppat.1006939.s011.tif]
